# Eco-friendly silver nanoparticles from garlic: a novel therapeutic approach for treating *Escherichia fergusonii* wound infections

**DOI:** 10.3389/fcimb.2025.1604507

**Published:** 2025-06-30

**Authors:** Sozan M. Abdelkhalig, Arwa Gamal Ali, Mohamed Farouk Ghaly, Nada K. Alharbi, Maha Alharbi, Mahmoud M. Bendary, Amira I. Abousaty

**Affiliations:** ^1^ Department of Basic Medical Sciences, College of Medicine, AlMaarefa University, Riyadh, Saudi Arabia; ^2^ Botany and Microbiology Department, Faculty of Science, Zagazig University, Zagazig, Egypt; ^3^ Department of Biology, College of Science, Princess Nourah bint Abdulrahman University, Riyadh, Saudi Arabia; ^4^ Department of Microbiology and Immunology, Faculty of Pharmacy, Port Said University, Port Said, Egypt

**Keywords:** wound infections, MDR, AgNPs, garlic extract, *E. fergusonii*, TEM

## Abstract

**Introduction:**

Complicated wound infections pose a significant challenge to patient recovery and healthcare systems, particularly due to the emergence of less common but highly resistant multidrug-resistant (MDR) pathogens that undermine the efficacy of conventional antibiotic therapies. This growing threat highlights the urgent need for innovative antimicrobial approaches.

**Methodology:**

In this study, we synthesized eco-friendly silver nanoparticles (AgNPs) using garlic extract to combat complicated wound infections caused by atypical MDR pathogens.

**Results:**

Genetic sequencing of 16S rRNA gene, aligned with phenotypic identification methods, confirmed that *Escherichia fergusonii* (*E. fergusonii*) as a significant atypical pathogen responsible for complicated wound infections, with a prevalence rate of 24% (12 out of 50 cases). Antimicrobial susceptibility testing revealed that all identified *E. fergusonii* strains exhibited MDR patterns. Garlic extract, analyzed using GC-MS and UPLC-ESI-MS/MS, identified sulfur-containing bioactive compounds such as allyl methyl trisulfide, dimethyl trisulfide, and allicin, which facilitated the biosynthesis of AgNPs. Stable, spherical AgNPs (15–20 nm) with strong antimicrobial properties were confirmed under optimal conditions (10 mL garlic extract, 40°C, pH 8.0). Their properties were validated using UV-Vis spectroscopy, XRD, and TEM. Antibacterial assays of AgNPs showed mean inhibition zones of 28±0.5 mm and MIC values of 100 µg/mL. TEM analysis revealed that AgNPs compromised bacterial membrane integrity, leading to structural damage, increased permeability, and leakage of intracellular contents. Simultaneously, they induced a concentration-dependent depletion of intracellular glutathione (GSH) in *E. fergusonii*, suggesting that both membrane disruption and oxidative stress synergistically contribute to bacterial cell lysis and death. A strong synergistic interaction was observed between AgNPs, used at a safe concentration below 50 µM as confirmed by cytotoxicity assays, and antibiotics such as ciprofloxacin, as evidenced by a fractional inhibitory concentration (FIC) index of 0.37. Time-kill assays demonstrated rapid bacterial eradication when AgNPs were combined with antibiotics such as ciprofloxacin.

**Conclusion:**

These findings underscore the promise of garlic-derived silver nanoparticles (AgNPs) as a fast-acting, eco-friendly option for treating complex wound infections caused by atypical multidrug-resistant (MDR) pathogens.

## Introduction

Complicated wound infections are a significant concern, particularly due to the presence of multidrug-resistant organisms. *Staphylococcus aureus*, including methicillin-resistant strains (MRSA), remains one of the most prevalent bacteria responsible for wound infections, often causing severe and persistent cases. *Pseudomonas aeruginosa*, known for its resistance to multiple antibiotics, is frequently isolated in more complicated or nosocomial infections, particularly in immunocompromised patients. *Klebsiella* spp., especially *Klebsiella pneumoniae*, is another common pathogen, notably in contaminated or dirty surgical wounds, contributing to both local and systemic infections. Additionally, *Acinetobacter* spp., notorious for surviving in hospital environments, has become a growing concern in abdominal surgeries, often complicating recovery and requiring targeted antimicrobial therapy. Among these pathogens, *Escherichia fergusonii* (*E. fergusonii*), a less well-known but emerging organism, has been increasingly identified as a cause of infection in abdominal wounds (Mahapatra et al., 2005; Adesina et al., 2019). These pathogens not only delay wound healing but also increase the risk of sepsis, leading to prolonged hospital stays and higher mortality rates. Effective management requires timely identification and appropriate antimicrobial therapy, with close monitoring of antibiotic resistance patterns, which vary across geographic regions and healthcare settings. Studies from various locations have confirmed the dominance of these organisms in abdominal wounds, as well as in urinary tract infections, bacteremia, diarrhea, and pleural infections, underlining the importance of infection control measures, including the careful administration of prophylactic antibiotics and enhanced surgical hygiene practices (Savini et al., 2008; Funke et al., 1993).

Silver nanoparticles (AgNPs) serve as effective antimicrobial agents, being incorporated into wound dressings and medical device coatings to prevent infections. AgNPs offer significant advantages over traditional antibiotics, especially in the fight against multidrug-resistant (MDR) infections. Unlike conventional antibiotics, which typically target specific bacterial functions, AgNPs exhibit broad-spectrum antimicrobial activity by disrupting bacterial cell walls, generating reactive oxygen species (ROS), and interfering with DNA replication, making them effective against a wide range of pathogens (Rai et al., 2009). Their unique ability to target multiple bacterial processes simultaneously reduces the likelihood of resistance development, a major issue with traditional antibiotics (Sondi and Salopek-Sondi, 2004). Furthermore, AgNPs’ small size and large surface area enhance their ability to penetrate bacterial cells more effectively, improving their antimicrobial efficacy (Rai et al., 2009). When combined with conventional antibiotics, AgNPs can also create a synergistic effect that overcomes resistance, making them a powerful tool for treating infections that have become resistant to standard treatments (Bahar and Ren, 2013).

Despite their widespread use, AgNPs have notable limitations that constrain their applications, particularly in medicine. One major concern is their potential cytotoxicity, as studies have shown that AgNPs can release silver ions, which may damage healthy cells and tissues, leading to oxidative stress and inflammatory responses in the body (Chen and Schluesener, 2008). Additionally, AgNPs may accumulate in organs such as the liver, lungs, and kidneys, raising concerns about long-term exposure and potential toxicity to human health (Marambio-Jones and Hoek, 2010). Environmental toxicity is also a concern, as the disposal of AgNPs can negatively impact aquatic and soil ecosystems (Fabrega et al., 2011). Due to these risks, AgNPs are generally preferred in applications where their powerful antimicrobial properties are crucial yet contained—such as in wound infections, where localized use minimizes systemic exposure. Their efficacy in preventing bacterial colonization and biofilm formation makes commercial silver-containing wound gels particularly valuable in wound care (Francolini et al., 2017), while reducing the need for broader exposure that might increase toxic risks. Alternatively, they can be used at low concentrations below cytotoxic levels in combination with commonly used antibiotics to enhance therapeutic outcomes.

AgNPs can be synthesized through various methods, primarily chemical, physical, and biological. The chemical reduction method is the most common, where silver ions (typically from silver nitrate) are reduced using agents like sodium borohydride or ascorbic acid, forming nanoparticles in solution. This method is widely favored for its simplicity, cost-effectiveness, and the ability to control particle size by adjusting factors such as pH, temperature, and stabilizers. Physical methods—such as evaporation-condensation and laser ablation—require specialized equipment and high energy inputs but yield high-purity AgNPs. Alternatively, biological synthesis (green synthesis) employs plant extracts or microorganisms as eco-friendly reducing agents, offering a sustainable and environmentally friendly option. Each method offers unique benefits depending on the intended application, from medical to industrial uses (Iravani, 2014; Mosallam et al., 2021).

However, green synthesis of AgNPs presents several advantages over chemical and physical methods. It relies on natural reducing agents such as plant extracts, bacteria, or fungi, eliminating the need for toxic chemicals and the high energy consumption associated with traditional techniques. This makes green synthesis a safer and more sustainable approach, with minimal environmental impact and reduced health hazards during production. Additionally, biological molecules in these natural sources often serve as stabilizers, enhancing nanoparticle stability and biocompatibility—key factors for medical and pharmaceutical applications. Green synthesis typically operates under mild conditions (room temperature and neutral pH), making it both simpler and more cost-effective (Mittal et al., 2013; Rai et al., 2009).

This study addresses the escalating issue of antimicrobial resistance in wound infections, with a particular focus on *E. fergusonii*, an emerging opportunistic pathogen. While *Staphylococcus aureus* and *Pseudomonas aeruginosa* are well-known wound pathogens, *E. fergusonii* has been increasingly implicated in various infections, including wounds, urinary tract infections, and bacteremia. Notably, it is frequently associated with multidrug resistance, including extended-spectrum β-lactamases and carbapenemases, posing significant treatment challenges. Despite its rising clinical relevance, *E. fergusonii* remains underexplored in the context of novel antimicrobial treatments. To address this gap, we propose a novel approach utilizing AgNPs synthesized via a green route using garlic (*Allium sativum*) extract. This method offers dual advantages: garlic acts as both a natural reducing and stabilizing agent in the synthesis of AgNPs and provides intrinsic antimicrobial properties. By using garlic’s dual functionality, our approach increases the antimicrobial potency of AgNPs while reducing the toxic effects and environmental concerns associated with chemical or radiation-based synthesis methods. (Prabhu and Poulose, 2012; Rai et al., 2009).

The synergistic action of garlic-based AgNPs is particularly promising against multidrug-resistant bacteria, as it targets multiple microbial pathways, thereby reducing the likelihood of resistance development and enhancing overall efficacy. Furthermore, combining these garlic-derived AgNPs with conventional antibiotics may restore the effectiveness of existing drugs against resistant strains, offering a sustainable and potent strategy for managing multidrug-resistant wound infections. While numerous studies have investigated AgNPs against common wound pathogens, few have applied green-synthesized nanoparticles to *E. fergusonii*, especially in the context of wound infections. To the best of our knowledge, this is the first study to explore the use of garlic-mediated AgNPs specifically against *E. fergusonii* isolated from complicated wound infections. This positions our research as a significant and novel contribution to both antimicrobial research and clinical practice, offering a targeted, eco-friendly approach to combat an emerging, multidrug-resistant pathogen.

## Materials and methods

### Identification of pathogenic bacteria in complicated wound Infections after abdominal surgery

#### Collection of clinical samples

One hundred and fifty wound swab samples were collected from wounds that exhibited clear signs of complication, such as persistent inflammation, delayed healing, or the presence of purulent discharge, at Zagazig University Hospitals, Egypt. After cleaning the infected sites, sterile cotton swabs were used to obtain specimens, which were then inoculated into brain heart infusion broth for enrichment and incubated for 24 hours at 37°C to promote pathogen growth. Subsequently, a loopful of the enriched culture was spread onto nutrient agar plates. The recovered colonies were identified using standard microbiological methods, including cultural characteristics, Gram staining, and biochemical tests (Madigan et al., 2018; Garcia, 2010).

During pathogen identification, when mixed isolates were encountered, laboratory protocols typically prioritized the isolates with larger colony counts, as these were more likely to represent the primary pathogen responsible for infection. Studies have indicated that isolates with colony counts exceeding 10^6^ CFU/mL are generally considered clinically significant and more likely to be associated with infection. This threshold is commonly used to differentiate between contamination and true infection, with lower counts often disregarded unless they exhibit distinct virulence factors or clear clinical relevance. Selecting the dominant pathogens is crucial in clinical settings to avoid unnecessary over-treatment and to focus on the most probable cause of infection (Bendy et al., 1964).

### Molecular identification

For further identification of the most prevalent isolate, which exhibited the highest MAR (Multiple Antibiotic Resistance) index, molecular techniques were employed—specifically 16S rRNA gene sequencing. This method provided a precise and reliable means of identifying the isolates at the species level, offering a more accurate understanding of their genetic composition and resistance profiles. DNA was extracted from the selected bacterial colonies. A fresh colony was mixed with 1 mL of sterile distilled water, vortexed, boiled for 10 minutes, incubated on ice for 5 minutes, and then centrifuged. The supernatant was transferred to an Eppendorf tube and stored at -20°C.

PCR amplification of the 16S rRNA gene was carried out using the forward primer [F27] (5’-AGAGTTTGATCCTGGCTCAG-3’) and reverse primer [R1492] (5’-GGTTACCTTGTTACGACTT-3’) (Weisburg et al., 1991). The amplification conditions were as follows: initial denaturation at 94°C for 10 minutes; 35 cycles of denaturation at 94°C for 30 seconds, annealing at 56°C for 1 minute, and extension at 72°C for 1 minute; followed by a final extension at 72°C for 10 minutes. PCR products were analyzed using 1% agarose gel electrophoresis, visualized under UV light after ethidium bromide staining (Sambrook and Russell, 2001), and purified using the GeneJET PCR Purification Kit. The purified PCR product was sequenced at GATC Biotech AG using an ABI 3730xl DNA sequencer, and the sequence was uploaded to NCBI (www.ncbi.nlm.nih.gov).

### Identification of multidrug-resistant isolates with the highest MAR index

The disk diffusion method was employed in triplicate to determine antimicrobial resistance patterns. The selected Bacterial cultures were grown on Mueller-Hinton agar plates (Lewis et al., 2025), and antimicrobial discs were carefully placed on the inoculated plates. nine different antimicrobial discs, representing various classes and concentrations, were used: Ampicillin (AMP, 10 µg), Amoxicillin/clavulanic acid (AMC, 10 µg), Tetracycline (TE, 30 µg), Vancomycin (VA, 30 µg), Ciprofloxacin (CIP, 5 µg), Ceftriaxone (CRO, 30 µg), Imipenem (IPM, 10 µg), Gentamicin (CN, 10 µg), and Amikacin (AK, 30 µg). The plates were kept at 4°C for 1 hour before incubation at 37°C for 24 hours. After incubation, the diameters of the inhibition zones were measured to assess bacterial susceptibility according to CLSI guidelines (Lewis et al., 2025).

Bacterial isolates showing resistance to at least one antimicrobial agent in three or more classes were classified as Multi-Drug Resistant (MDR) (Magiorakos et al., 2012). To evaluate the extent of resistance, the Multiple Antibiotic Resistance (MAR) index was calculated for the selected isolate by dividing the number of antibiotics to which the isolate was resistant by the total number tested. A higher MAR index indicates substantial antimicrobial exposure, often associated with high-risk environments, and provides important insights for infection control and antimicrobial stewardship (Mir et al., 2022).

### Green synthesis of silver nanoparticles

#### Preparation of the alcoholic garlic extract

A total of 30 grams of fresh garlic bulbs were used to prepare two separate extracts: one for the green synthesis of AgNPs, and the other for chemical analysis of bioactive compounds. The fresh garlic was powdered and subjected to extraction with 7 mL of 70% ethanol using a maceration technique. The ethanol was subsequently removed by concentrating the extract under reduced pressure at 40°C using a rotary evaporator. This process eliminated the ethanol, leaving behind a concentrated plant extract, which was stored at 4°C for future use (Sakkas and Papadopoulou, 2017).

The concentrated extract was dissolved in DMSO to achieve a final concentration of 50 µg/mL, transferred to a sterile flask, and stored at 4°C until use (Ghaly et al., 2023). To preserve the stability of sulfur-containing compounds, such as allicin, all extraction steps were conducted under cold conditions (4–8°C), protected from light, and processed immediately after preparation. The extract designated for LC–MS analysis was filtered, briefly stored at –20°C, and analyzed within 24 hours to minimize degradation; however, the other sample was used directly in the synthesis of AgNPs.

### Isolation, purification, and identification of active components in garlic extract using gas chromatography–mass spectrometry analysis

To identify volatile bioactive compounds in the partially purified garlic extract, gas chromatography–mass spectrometry (GC-MS) analysis was performed following established protocols (Sparkman, 2005). The extract, previously shown to exhibit antibacterial activity at an Rf value of 9 via thin-layer chromatography (TLC), was subjected to chromatographic profiling (Shang et al., 2024). Samples were analyzed using a GC-MS system equipped with a capillary column (e.g., HP-5MS, 30 m × 0.25 mm × 0.25 μm film thickness). The injector was maintained at 250°C, with helium as the carrier gas at a constant flow rate of 1.0 mL/min. The oven temperature was initially set at 50°C for 2 minutes, ramped at 10°C/min to 280°C, and held for 5 minutes. The sample (1 μL) was injected in splitless mode. The mass spectrometer operated in electron ionization (EI) mode at 70 eV with a scan range of m/z 50–500 (Lanzotti, 2006). Chromatograms were analyzed using integrated spectral libraries (e.g., NIST, Wiley) to identify compounds based on retention time and mass fragmentation patterns. (Ankri and Mirelman, 1999). Identification of sulfur-containing compounds, such as allicin, was confirmed by comparison with a commercially available synthetic standard, matching both the retention factor and the mass-to-charge ratio (m/z), thereby validating the presence of allicin in the sample.

### Ultra-performance liquid chromatography-electrospray ionization-mass spectrometry

The bioactive compounds identified on the TLC plate (at an Rf value of 0.9) were further analyzed using liquid chromatography–mass spectrometry (LC–MS). The analysis was conducted with a Thermo Scientific LCQ Deca mass spectrometer equipped with a Hypersil Gold aQ C18 column and an electrospray ionization (ESI) source operating in positive ion mode (Oyaluna et al., 2024). The mobile phases consisted of 0.1% formic acid in water (A) and acetonitrile with 0.1% formic acid (B). Gradient elution was performed, beginning with 2% mobile phase B and increasing to 98% over 30 minutes, at a flow rate of 0.2 mL/min, for a total run time of 40 minutes. The chemical identities of the resolved compounds were determined by comparing their mass spectral patterns and retention times with entries in the NIST mass spectral library.

### Synthesis of silver nanoparticles from garlic extract

An aqueous solution of silver nitrate (200 µg/mL) was prepared for the green synthesis of AgNPs. To initiate the reduction of silver ions (Ag^+^), 10 mL of garlic extract was mixed with 10 mL of the silver nitrate solution. The reaction mixture was then exposed to sunlight for 24 hours, during which a gradual color change from colorless to dark brown was observed, indicating the formation of AgNPs. This green synthesis approach is both environmentally friendly and effective, employing natural reducing agents present in garlic extract to drive the reduction process (Sarangi et al., 2024).

### Optimization of silver nanoparticles synthesis

To optimize the synthesis of AgNPs, different concentrations of garlic extract, temperatures, and pH levels were evaluated. AgNPs were synthesized by mixing 10 mL of silver nitrate solution (200 µg/mL) with freshly prepared garlic extract of the same concentration. The initial synthesis involved adding 5 mL of garlic extract to the AgNO_3_ solution at room temperature, followed by continuous stirring for 30–60 minutes. A color change from colorless to yellowish-brown was observed, indicating the formation of nanoparticles due to the reduction of silver ions (Ag^+^).

Optimization was conducted by varying garlic extract volumes (10–40 mL), reaction temperatures (20–60°C), and pH values (6.0–9.0) to determine the most favorable conditions for producing stable nanoparticles. Characterization of the synthesized AgNPs was carried out using UV–Visible spectroscopy (showing characteristic absorption peaks around 400–450 nm), transmission electron microscopy (TEM), and X-ray diffraction (XRD) analysis. Antimicrobial activity was assessed using the agar diffusion method. The final nanoparticle suspension was stored at 4°C for further analysis (Singh et al., 2015; Ahmed et al., 2016a; Mittal et al., 2013)

### Stability assessment in simulated wound fluid

To evaluate the colloidal stability of biosynthesized AgNPs under physiological conditions, the nanoparticles were incubated in simulated wound fluid (SWF). SWF was prepared by mixing 50% (v/v) fetal bovine serum (FBS) with phosphate-buffered saline (PBS, pH 7.4), mimicking the protein-rich and ionic environment of wound exudate, as described in previous studies (Rozman et al., 2020). AgNPs were diluted in SWF to a final concentration of 50 µg/mL and incubated at 37 °C for 72 hours. Stability was assessed at 0, 24, 48, and 72 hours using UV–Visible spectroscopy (300–700 nm) to monitor shifts in the surface plasmon resonance (SPR) peak. Zeta potential measurements were performed using a Zetasizer Nano ZS (Malvern Instruments, UK), following previously published **protocols** (Massironi et al., 2022; Rónavári et al., 2021). Visual observations were also recorded for signs of nanoparticle sedimentation or aggregation, which could indicate destabilization under biorelevant conditions (Königs et al., 2015).

### Characteristics of biosynthesized silver nanoparticles

#### Ultraviolet spectroscopy

The biosynthesis of AgNPs using garlic extract at various concentrations was monitored using UV–visible spectroscopy. Measurements were taken over time with a double-beam spectrophotometer (Shimadzu UV-1650 PC, Osaka, Japan) across a wavelength range of 190–790 nm. Characteristic peaks in the UV–Vis spectra confirmed the formation of AgNPs (Ahmed et al., 2016a).

### X-ray diffraction of the silver nanoparticles

X-ray diffraction (XRD) analysis (Shimadzu XD-3A, Japan) was employed to determine the crystalline nature and grain size of AgNPs biosynthesized from garlic extract. The particle size of the nanoparticles was calculated using Scherrer’s equation:

D = Kλ/βcosθ, where D represents the average crystallite size, β is the line broadening in radians (full width at half maximum of the peak), λ is the X-ray wavelength, θ is the Bragg angle, and K is a constant (shape factor, typically 0.94). This equation provides an estimate of nanoparticle size based on the broadening of diffraction peaks observed in the XRD pattern (Singh et al., 2015; Mittal et al., 2013).

### Transmission electron microscopy analysis primarily

Transmission Electron Microscopy (TEM) was utilized to validate the synthesis method for AgNPs and to assess their shape. TEM analysis was conducted at the Electron Microscopy Unit of the National Research Center, following the previous published protocol (Vanlalveni et al. (2021). This technique provided detailed insights into the morphology and structural characteristics of the AgNPs. TEM is commonly used to examine the size, shape, and crystallinity of nanoparticles, offering high-resolution imaging crucial for confirming their formation and structure (Kumari et al., 2017).

### Antimicrobial effect of silver nanoparticles

The antibacterial potential of AgNPs was assessed using the agar well diffusion method. The MDR bacterial culture with the highest MAR index, standardized to a 0.5 McFarland turbidity standard, were evenly spread across sterile Mueller-Hinton agar plates to ensure uniform bacterial distribution. AgNPs, suspended in DMSO at a concentration of 10 mg/mL, were introduced into wells drilled in the agar, with 100 µL of the nanoparticle solution added to each well. The plates were then incubated at 37°C for 24 hours to allow bacterial growth and interaction with the nanoparticles. After incubation, the inhibition zones around each well were measured in millimeters to evaluate the antimicrobial activity of the AgNPs. Positive and negative control wells contained an antimicrobial to which the tested isolate was susceptible, and DMSO, respectively, were included for comparison (Mosallam et al., 2021; Lewis et al., 2025).

### Determination of minimum inhibitory concentration of silver nanoparticles

The minimum inhibitory concentration (MIC) of the synthesis AgNPs against the MDR bacterial culture with the highest MAR index was determined using the broth microdilution method. The nanoparticles were initially dissolved in dimethyl sulfoxide (DMSO) to achieve final concentrations ranging from 10 µg/mL to 200 µg/mL. Each dilution was added to a 96-well plate, along with appropriate negative and positive controls: culture broth with DMSO and culture broth with an antimicrobial to which the tested isolates were sensitive respectively. Each well was inoculated with 5 µL of bacterial suspension, adjusted to a concentration of 10^5^ CFU/mL, to assess the antimicrobial effect of the AgNPs (Eloff, 1998; Mosallam et al., 2023 **);.** Experiments were conducted in triplicate, with microdilution trays incubated at 37°C for 18 h. MIC was determined by measuring optical density using a spectrophotometer, based on ELISA principles for bacterial inhibition. The MIC was the lowest concentration of AgNPs that fully inhibited growth (Valgas et al., 2007).

### Assessment of ROS generation via detection of reduced glutathione levels

To biochemically detect reduced glutathione (GSH) levels in bacteria treated with AgNPs, the Ellman’s reagent (DTNB) assay was employed. This method has been widely used for quantifying thiol groups in biological samples (Ellman, 1959). Bacterial cultures were first grown to mid-log phase and then exposed to AgNPs. Following exposure, the cells were harvested by centrifugation and washed with cold phosphate-buffered saline (PBS). The cell pellets were resuspended in 5% sulfosalicylic acid (SSA) to precipitate proteins and release intracellular GSH, followed by incubation on ice for 15 minutes. After centrifugation, the supernatant containing GSH was collected. For the assay, 100 µL of the supernatant was mixed with 900 µL of DTNB reagent (0.6 mM DTNB in 0.1 M phosphate buffer, pH 7.4) and incubated at room temperature for 5–10 minutes. The yellow-colored product, TNB, formed from the reaction between GSH and DTNB, was then measured spectrophotometrically at 412 nm. A standard curve prepared using known GSH concentrations was used to quantify GSH levels, providing an indication of oxidative stress induced by AgNP exposure (Rahman et al., 2006; Aebi, 1984).

### *In vitro* cytotoxicity assessment

To determine the safe concentration range of AgNPs, their cytotoxic potential was evaluated *in vitro* using fibroblast cells. Fibroblasts were cultured in Dulbecco’s Modified Eagle Medium (DMEM) supplemented with 10% fetal bovine serum (FBS) and 1% penicillin–streptomycin, and maintained under standard conditions (37°C, 5% CO_2_, humidified atmosphere). Cells were seeded into 96-well plates at a density of 5 × 10^4^ cells per well and incubated overnight to allow for adherence. The nanoparticle formulation was applied at varying concentrations, and cell viability was assessed after 24 and 48 hours of exposure using the 3-(4,5-dimethylthiazol-2-yl)-2,5-diphenyltetrazolium bromide (MTT) assay. Absorbance was measured at 570 nm using a microplate reader, and the percentage of viable cells was calculated relative to untreated controls. These results were used to determine the cytotoxic threshold and the optimal non-toxic concentration of the AgNPs (Zhang et al., 2010; Arora et al., 2009).

### Evaluation of the combined antibacterial activity of silver nanoparticles and most common antibiotics “ciprofloxacin” against *E. fergusonii*

To investigate the interaction between AgNPs and the commonly used antibiotic ciprofloxacin, a checkerboard assay was employed. This method allows for the determination of the fractional inhibitory concentration (FIC) index, which quantifies the interaction between two antimicrobial agents. Serial two-fold dilutions of both AgNPs and ciprofloxacin were prepared in triplicate across a 96-well microtiter plate to cover a range of concentrations for each compound. Bacterial suspensions of the tested isolates were then inoculated into the wells and incubated at 37°C for 18–24 hours. The minimum inhibitory concentrations (MICs) of each agent, both individually and in combination, were determined in triplicate. The FIC index was calculated using the following equation: FIC index = (MIC of AgNPs in combination/MIC of AgNPs alone) + (MIC of ciprofloxacin in combination/MIC of ciprofloxacin alone). Interpretation of the FIC index values followed established guidelines: ≤0.5 indicated a synergistic effect, >0.5 to ≤1.0 suggested an additive effect, and values >1.0 implied antagonism. This method provides a valuable tool for optimizing antimicrobial therapy by identifying combinations that enhance or hinder antibacterial efficacy (Odds, 2003; Tängdén, 2014).

### Mode of action of biosynthesis nanoparticles by transmission electron microscope

TEM was employed to examine the treated isolate with AgNPs, which were incubated for 24 hours. The analysis was performed at the Electron Microscopy Unit at Mansoura University. The samples were imaged using a JEOL-JEM-2100 transmission electron microscope. TEM was used to observe and comp Transmission electron microscopy (TEM) was employed to examine bacterial isolates treated with AgNPs following a 24-hour incubation. The analysis was conducted at the Electron Microscopy Unit of Mansoura University using a JEOL JEM-2100 transmission electron microscope. TEM imaging was used to observe and compare ultrastructural changes in AgNP-treated bacterial cells with those of untreated control cells (Kumari et al., 2017).

### Time-kill curve experiments

The time-kill assay was conducted to evaluate the bactericidal activity of AgNPs, ciprofloxacin, and their combination against *E. fergusonii*. The bacterial strain was cultured in Mueller-Hinton Broth (MHB) to an initial inoculum of approximately 1 × 10^6^ CFU/mL. Treatments included AgNPs at their minimum inhibitory concentration (MIC) and sub-MIC levels, ciprofloxacin at MIC and sub-MIC levels, and a combination of AgNPs at a non-cytotoxic concentration with ciprofloxacin at sub-MIC. A drug-free culture served as the negative control for comparison. Cultures were incubated at 37°C with shaking, and aliquots were collected at 2-hour intervals up to 24 hours. At each time point, serial dilutions were prepared in sterile saline, and appropriate dilutions were plated on Mueller-Hinton agar. Plates were incubated at 37°C for 18–24 hours, and colony-forming units (CFU) were counted to determine viable bacterial counts. Results were expressed as log_10_ CFU/mL and plotted against time (Montero et al., 2021).

### Statistical analysis

All experiments were conducted in triplicate, and results are expressed as the mean ± standard deviation (SD). Statistical analyses were performed using GraphPad Prism version 9.0. To evaluate significant differences among treatment groups (e.g., inhibition zone diameters), a one-way analysis of variance (ANOVA) was conducted. When ANOVA indicated statistical significance (p < 0.05), Tukey’s multiple comparisons *post hoc* test was applied to identify pairwise differences between groups. A p-value less than 0.05 was considered statistically significant. Graphs were generated using GraphPad Prism, with error bars representing standard deviations.

## Results

### Phenotypic characterization of bacterial isolates in surgical site infections

In our study, we identified various pathogens responsible for complicated wound infections using standard microbiological methods, including culture characteristics, Gram staining, and a range of biochemical tests. From 150 wound swab samples, 50 bacterial isolates were obtained. *E. fergusonii* was identified as a newly emerging wound pathogen, with a prevalence rate of 24% (12/50). *E. fergusonii* appeared as pink colonies on MacConkey agar due to lactose fermentation, exhibited Gram-negative rod morphology, and was confirmed through positive indole, lysine decarboxylase, and methyl red tests. It was further distinguished by positive arginine dihydrolase and citrate utilization results.

### Molecular confirmation and characterization of selected bacterial isolates

The DNA sequences obtained from PCR of 16S RrNA genes were compared with published sequences using the BLAST tool (http://www.ncbi.nlm.nih.gov/blast). The purified PCR products were analyzed to determine its similarity to sequences available in GenBank. Molecular identification of the investigated *E. fergusonii* isolates (n = 12) yielded identical sequences. Sequence alignment showed over 96% nucleotide identity with previously published data in GenBank, confirming the selected isolates and consistent with phenotypic identification. The sequences were submitted to GenBank under the accession number OP57747.

### Characterization of multidrug resistance and MAR indices in clinical isolates

Notably, all *E. fergusonii* isolates exhibited multidrug-resistant (MDR) patterns, showing resistance to at least three antibiotics from different antimicrobial classes. The multiple antibiotic resistance (MAR) indices of the clinical isolates ranged from 0.56 to 0.78, indicating moderate to high resistance levels. Based on these results, we selected the ten isolates (N=10) with the highest MAR index (0.78) for further analysis and excluded two isolates with moderate MAR indices.

### Chemical analysis of purified garlic extract using instrumental analysis

GC-MS analysis of the partially purified garlic extract revealed a complex chemical profile consisting of numerous volatile components as shown in **Supplementary Figure S1**. A total of 48 peaks were detected across two chromatograms, with compounds eluting between 1.54 and 31.58 minutes. The most prominent peaks appeared at retention times of 21.05, 15.11, and 6.88 minutes in the first chromatogram, contributing 21.93%, 10.55%, and 9.01% of the total area, respectively. In the second chromatogram, major peaks were observed at 31.37 minutes (32.78%), 25.41 minutes (10.32%), and 30.37 minutes (8.24%). These peaks indicate the presence of dominant volatile constituents within the extract. A spot detected at Rf value 9 on thin-layer chromatography (TLC) exhibited strong antibacterial activity against selected multidrug-resistant (MDR) bacterial strains. This bioactive fraction was subjected to GC-MS analysis, which allowed identification of key antimicrobial compounds.

UPLC-ESI-MS/MS analysis of the bioactive fraction identified at Rf = 0.9 on the TLC plate revealed the presence of several distinct compounds. The chromatographic separation produced well-resolved peaks, and mass spectral data acquired in positive ion mode allowed accurate identification of the constituents. By comparing the mass spectra and retention times with the NIST library, multiple bioactive molecules were confirmed, supporting the chemical complexity and antimicrobial potential of the active garlic extract fraction. Based on retention time, peak area, and comparison with reference data, three major sulfur-containing compounds were identified: allyl methyl trisulfide (C_4_H8S_3_, MW 152.3), dimethyl trisulfide (C_2_H_2_S_3_, MW 126.3), and allicin (C_2_H_10_S_2_, MW 162.3) as shown in **Table 1**. These compounds are known for their broad-spectrum antimicrobial properties and are likely responsible for the observed antibacterial activity. The results confirm that sulfur-rich volatile constituents are predominant in the garlic extract and contribute to its therapeutic potential.

**Table 1:** Chemical component and chemical structure of purified garlic extract.

Compound	MW	RT	Peak	Molecular formula	Structure
Ally methyl trisulfide	152.3	31.29	46	C_4_H_8_S_3_	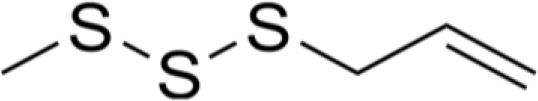
Dimethyl trisulphide	126.3	30.87	42	C_2_H_6_S_3_	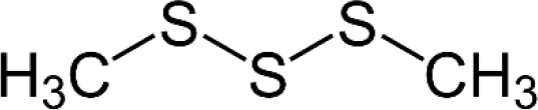
Allicin	162.3	6.88	5	C_6_H_10_S_2_	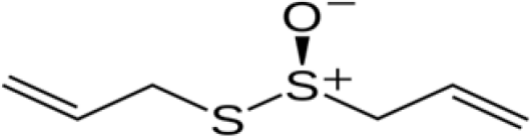

### Biosynthesis and characterization of silver nanoparticles using garlic extract

AgNPs were successfully synthesized using garlic extract, evidenced by a distinct color change from white to dark brown, indicating the reduction of Ag^+^ ions. Optimization revealed that garlic extract volume, temperature, and pH significantly influenced nanoparticle formation, stability, and size. The most stable and uniformly sized AgNPs, with strong antimicrobial activity, were obtained at 10 mL extract volume, 40°C, and pH 8.0. At this condition, the reaction mixture consistently turned yellow-brown within 30–45 minutes. At higher temperatures (>40°C), although the reaction rate increased, nanoparticle aggregation also intensified, resulting in polydisperse and less stable colloids with reduced antimicrobial efficacy. Similarly, more alkaline conditions (pH 9–10) accelerated nucleation but produced irregular and unstable nanoparticles, likely due to unregulated reduction kinetics. These conditions led to inconsistent morphology and poor colloidal stability. In contrast, pH 8.0 provided a balanced environment that moderated the reduction rate, enabling controlled nucleation and growth. This favored the formation of uniform, stable, and bioactive AgNPs, making it the optimal condition for synthesis.

**Figure 1:**
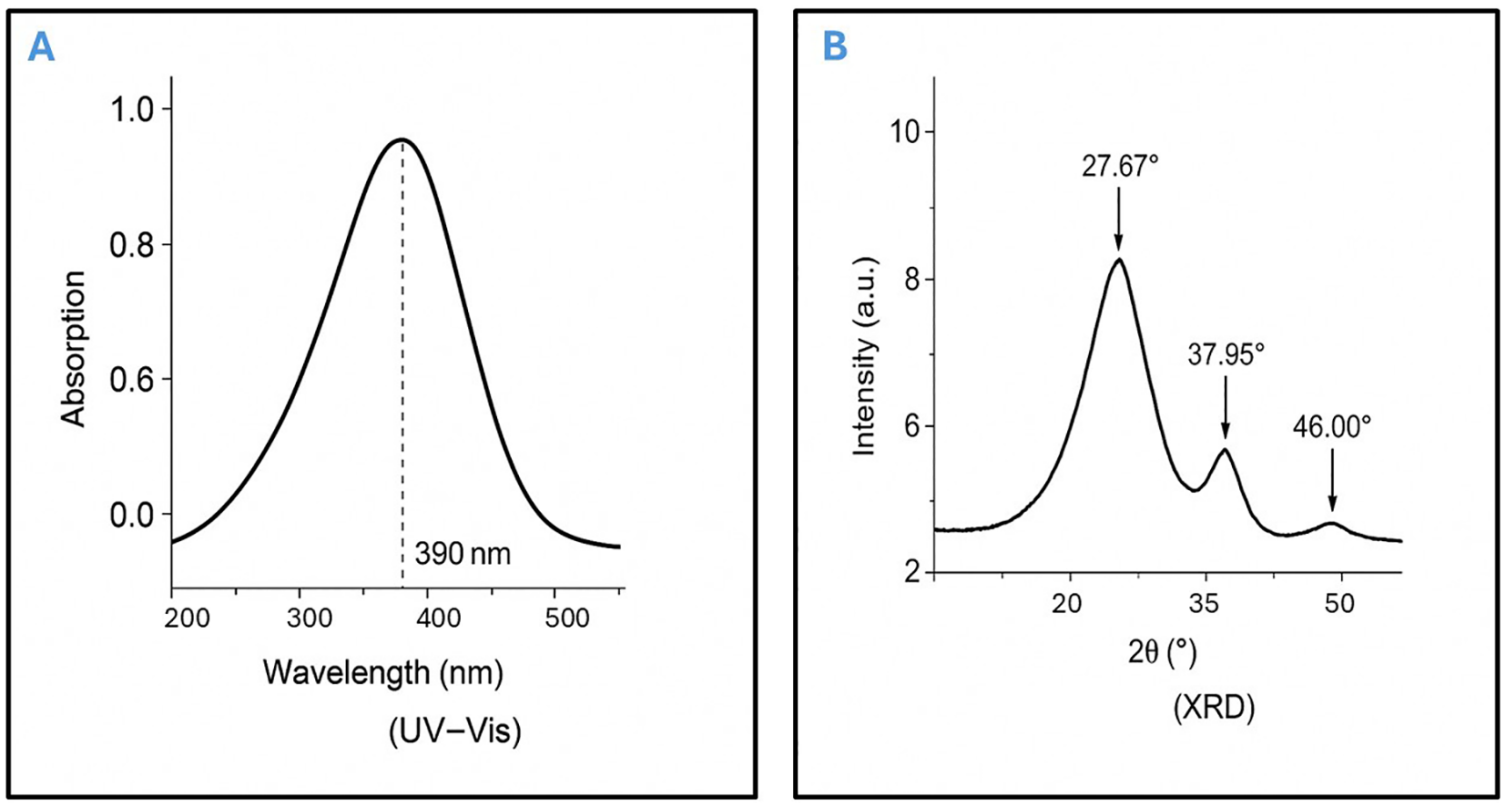
Characterization of garlic-mediated AgNPs by UV–Vis spectroscopy and X-ray diffraction. **(A)** UV–Vis Spectroscopy of Synthesized AgNPs, the UV–Visible absorption spectrum displays a characteristic surface plasmon resonance (SPR) peak at approximately 390 nm, confirming the formation of well-dispersed silver nanoparticles (AgNPs). **(B)** X-ray Diffraction (XRD) Pattern of AgNPs, the XRD profile reveals distinct diffraction peaks at 27.67°, 37.95°, and 46.00°, indicating the crystalline nature of the synthesized AgNPs and corresponding to the face-centered cubic (FCC) structure of elemental silver.

**Figure 2:**
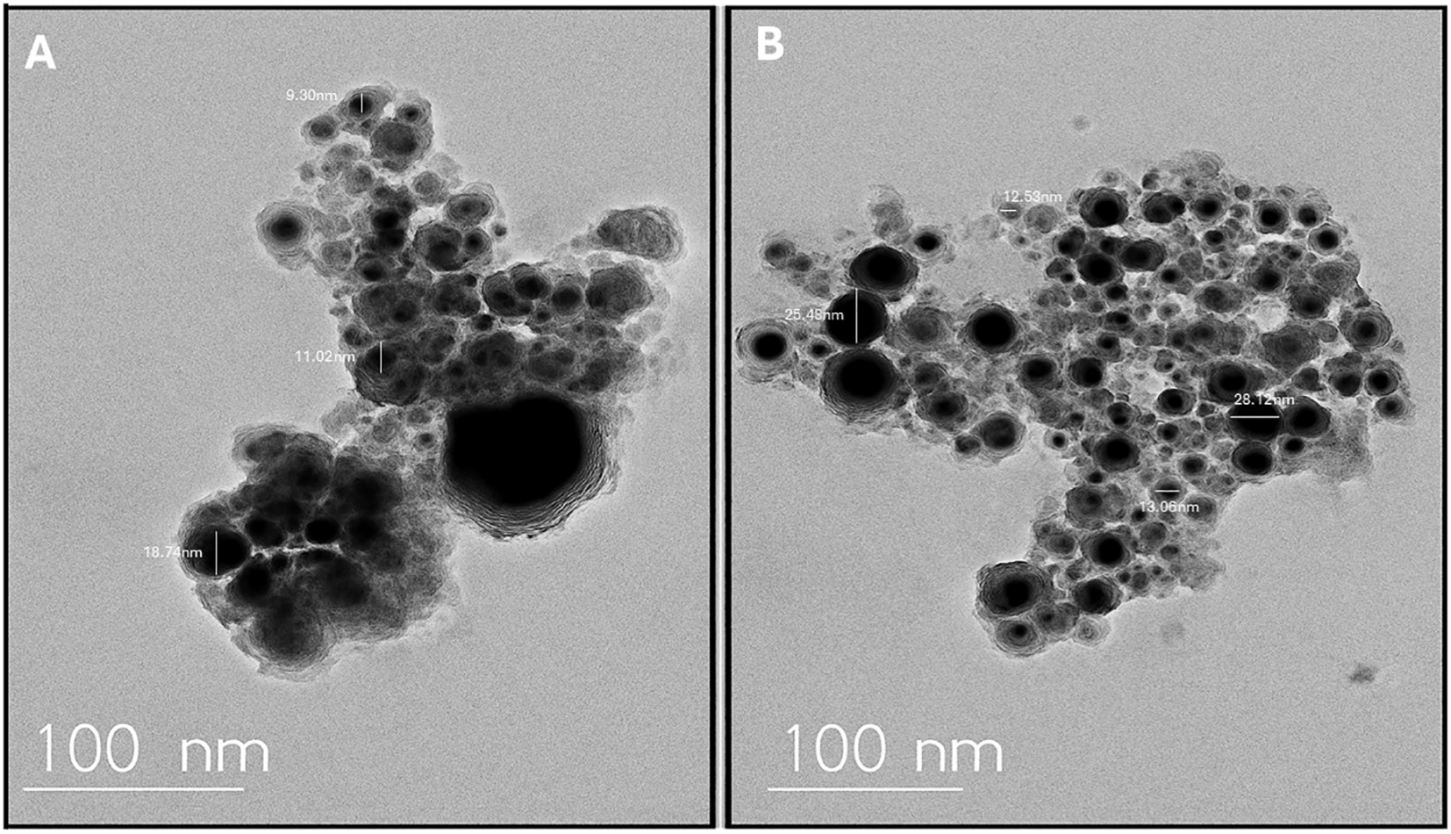
Transmission electron microscopy (TEM) image showing the morphology and size distribution of silver nanoparticles (AgNPs) synthesized using garlic extract. **(A)** Depicts the silver nanoparticles with a spherical shape and consistent size, confirming effective synthesis. **(B)** Presents the size distribution profile of the AgNPs, highlighting their uniformity and supporting the structural consistency of the sample.

Moreover, UV-Visible spectroscopy further confirmed nanoparticle formation, showing a characteristic absorption peak at approximately 390 nm, indicative of well-dispersed AgNPs. the concentration of Ag+ ions was the primary factor significantly affecting the biosynthesis of AgNPs, as confirmed by the corresponding absorption spectra, which showed a prominent peak at 390 nm (**Figure 1A**). X-ray diffraction (XRD) analysis verified the purity and crystalline structure of the synthesized AgNPs, showing prominent diffraction peaks at 27.67°, 32.08°, 37.95°, and 46.00°, confirming well-defined crystalline AgNPs (**Figure 1B**). Transmission electron microscopy (TEM) analyses showed that nanoparticles synthesized under optimized conditions had an average size of 15–20 nm and were spherical in shape. TEM images revealed a narrow size distribution, with most particles falling within the 4.21 to 22.67 nm range, suggesting a relatively uniform size (**Figures 2A, B**). These optimized parameters (10 mL garlic extract, 40°C, and pH 8.0) produced AgNPs with desirable size, stability, and antimicrobial properties, suggesting that these conditions are optimal for efficient synthesis and potential applications.

**Figure 3:**
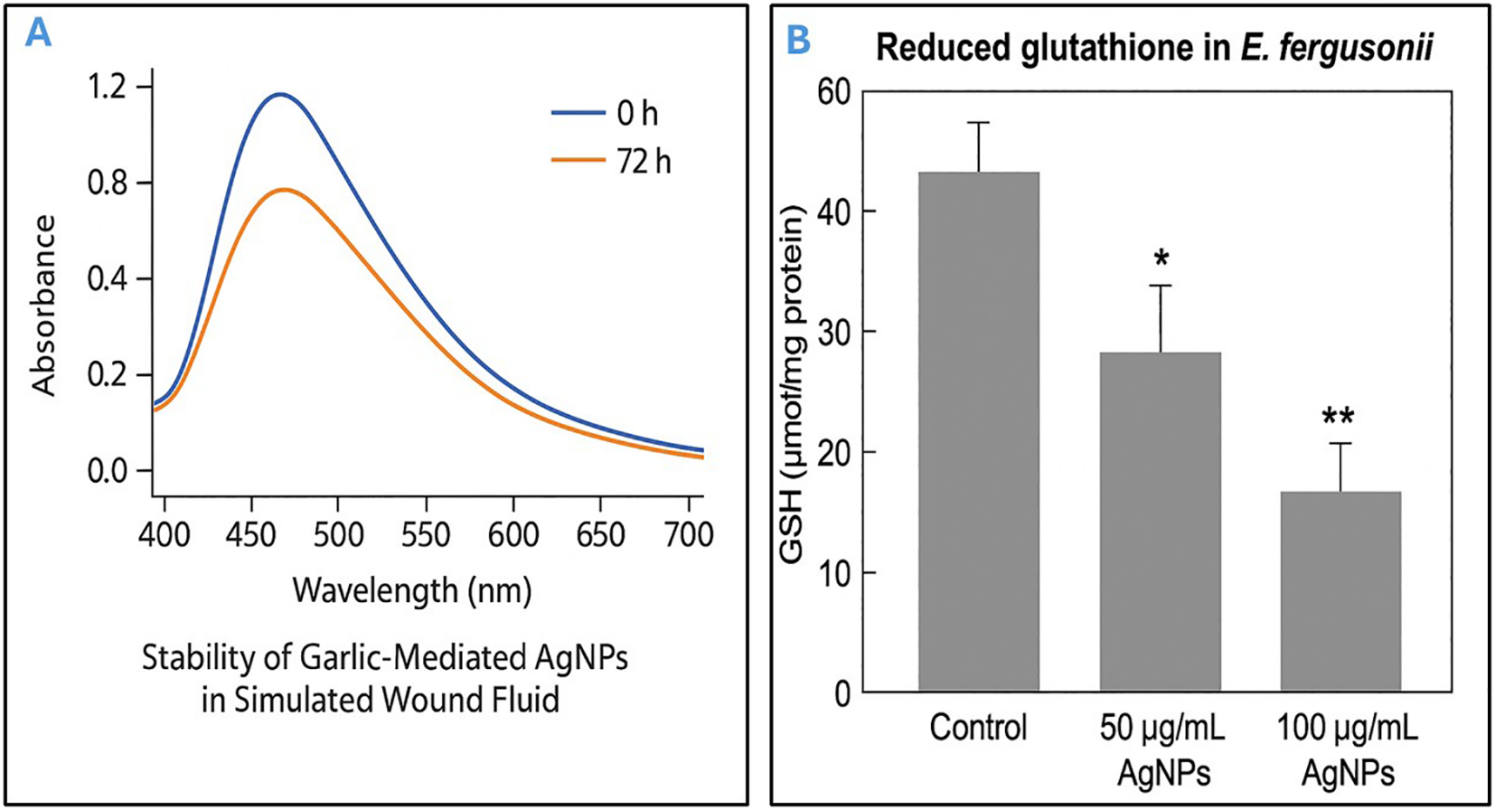
Evaluation of AgNP stability in simulated wound fluid and associated oxidative stress in *Escherichia fergusonii*. **(A)** Stability of garlic-mediated silver nanoparticles (AgNPs) in simulated wound fluid (SWF) at 37°C over a 72-hour period, as determined by UV–Visible spectroscopy. The surface plasmon resonance (SPR) peak remained near 450 nm with minimal spectral shift and reduction in absorbance, indicating good colloidal stability and minimal aggregation. **(B)** Effect of AgNPs on reduced glutathione (GSH) levels in *Escherichia fergusonii*. Exposure to AgNPs at 50 and 100 µg/mL significantly reduced intracellular GSH concentrations compared to the untreated control. Data are expressed as mean ± standard deviation (SD). *p* < 0.05 (*), *p* < 0.01 (**), indicating statistical significance relative to control.

Importantly, garlic-mediated AgNPs demonstrated excellent stability in simulated wound fluid (SWF) over a 72-hour incubation period at 37°C. UV–Vis spectra showed minimal shift in the surface plasmon resonance (SPR) peak between 0 h and 72 h, indicating preserved nanoparticle dispersion and morphology. No visible aggregation or sedimentation was observed during the incubation. Zeta potential measurements remained consistently negative, with values ranging from −28.6 mV to −26.9 mV across the time points, confirming stable electrostatic repulsion and colloidal integrity (**Figure 3A**). These findings suggest that the phytochemical capping agents from *Allium sativum* extract effectively stabilized the AgNPs even under physiologically relevant conditions.

### Antimicrobial effect of silver nanoparticles

The antimicrobial activity of AgNPs synthesized using garlic extract was assessed against selected *E. fergusonii* isolates (N=10) with high MAR indices. The inhibition zone diameters were measured, with a mean value of 28 ± 0.5 mm; however, the MIC for most tested isolates was 100 µg/mL. These results suggest that AgNPs synthesized using garlic extract possess significant antibacterial potential, with varying efficacy against different MDR pathogens.

### Results of oxidative stress analysis via ROS assay

Following treatment with AgNPs, a dose-dependent decrease in reduced glutathione (GSH) levels was observed in *E. fergusonii* compared to untreated controls. In the control group, intracellular GSH concentration was measured at 52.3 ± 3.1 µmol/mg protein. Upon exposure to 50 µg/mL and 100 µg/mL of AgNPs, GSH levels significantly dropped to 31.7 ± 2.5 µmol/mg and 18.2 ± 1.8 µmol/mg protein, respectively (p < 0.01) (**Figure 3B**). This decline indicates a marked oxidative stress response, suggesting that AgNPs induce GSH depletion in a concentration-dependent manner. The results are consistent with elevated oxidative damage and support the hypothesis that AgNPs exert their bactericidal activity, to some extent, via oxidative stress mechanisms.

### *In vitro* cytotoxicity assay

**Figure 4:**
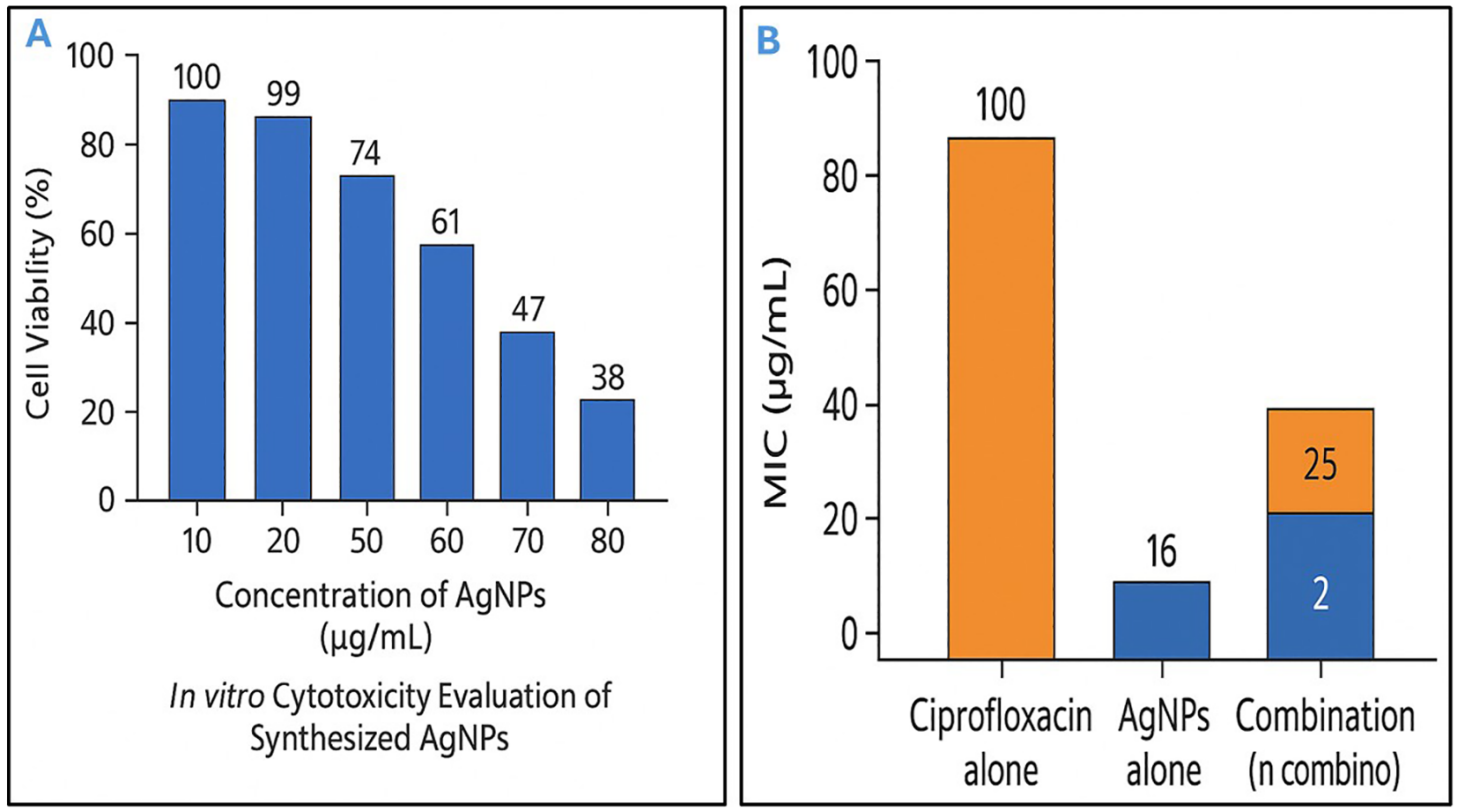
Dual characterization of silver nanoparticles: safe concentrations and antibacterial synergy with conventional antibiotic. **(A)** *In vitro* cytotoxicity evaluation of biosynthesized silver nanoparticles (AgNPs) against normal human dermal fibroblasts. Cell viability was assessed after 24 hours of exposure to increasing concentrations of AgNPs (10–80 µg/mL), showing a dose-dependent reduction in viability. Concentrations below 50 µg/mL maintained over 60% cell viability, indicating relatively low cytotoxicity. **(B)** Antibacterial activity of ciprofloxacin, AgNPs, and their combination against *Escherichia fergusonii*. Minimum inhibitory concentration (MIC) values are shown. Ciprofloxacin and AgNPs alone exhibited MICs of 16 µg/mL and 100 µg/mL, respectively. The combination reduced MICs to 2 µg/mL (ciprofloxacin) and 25 µg/mL (AgNPs), demonstrating a synergistic effect.

*In vitro* cytotoxicity evaluation of the synthesized AgNPs revealed a dose-dependent response in normal human dermal fibroblasts. Cell viability remained above 90% at concentrations up to 30 µg/mL, while exposure to 50 µg/mL resulted in a moderate reduction to approximately 60% viability after 24 hours, indicating a clear cytotoxic threshold. Therefore, concentrations below 50 µg/mL were unlikely to have caused significant harm to the fibroblast cells, as they induced less than 50% of the maximal cytotoxic effect (**Figure 4A**). As a result, these lower concentrations were considered relatively safe for cellular exposure during the experimental conditions.

### Assessment of synergistic antibacterial activity of AgNPs and ciprofloxacin against *E. fergusonii*

The MIC of AgNPs alone against *E. fergusonii* was 100 µg/mL, while ciprofloxacin alone exhibited an MIC of 16 µg/mL. When used in combination, the MICs were significantly reduced to 25 µg/mL for AgNPs and 2 µg/mL for ciprofloxacin (**Figure 4B**). These findings were consistent in 80% of the selected *E. fergusonii* isolates (N=10) with high multiple antibiotic resistance (MAR) indices. This notable reduction in MIC values indicated a potentiation of antibacterial activity through co-administration. The FIC index for this combination was calculated to be 0.37, which was well below the synergy threshold of 0.5, thereby demonstrating a synergistic interaction between AgNPs and ciprofloxacin. Therefore, the combined use of AgNPs and ciprofloxacin exerted a more potent antibacterial effect against *E. fergusonii* than either agent used alone. Such synergy not only enhanced bacterial growth inhibition but also implied the potential for reducing the required concentrations of each antimicrobial agent, which could help minimize cytotoxicity and delay the emergence of resistance.

### Mode of action of biosynthesis nanoparticles by transmission electron microscope

**Figure 5:**
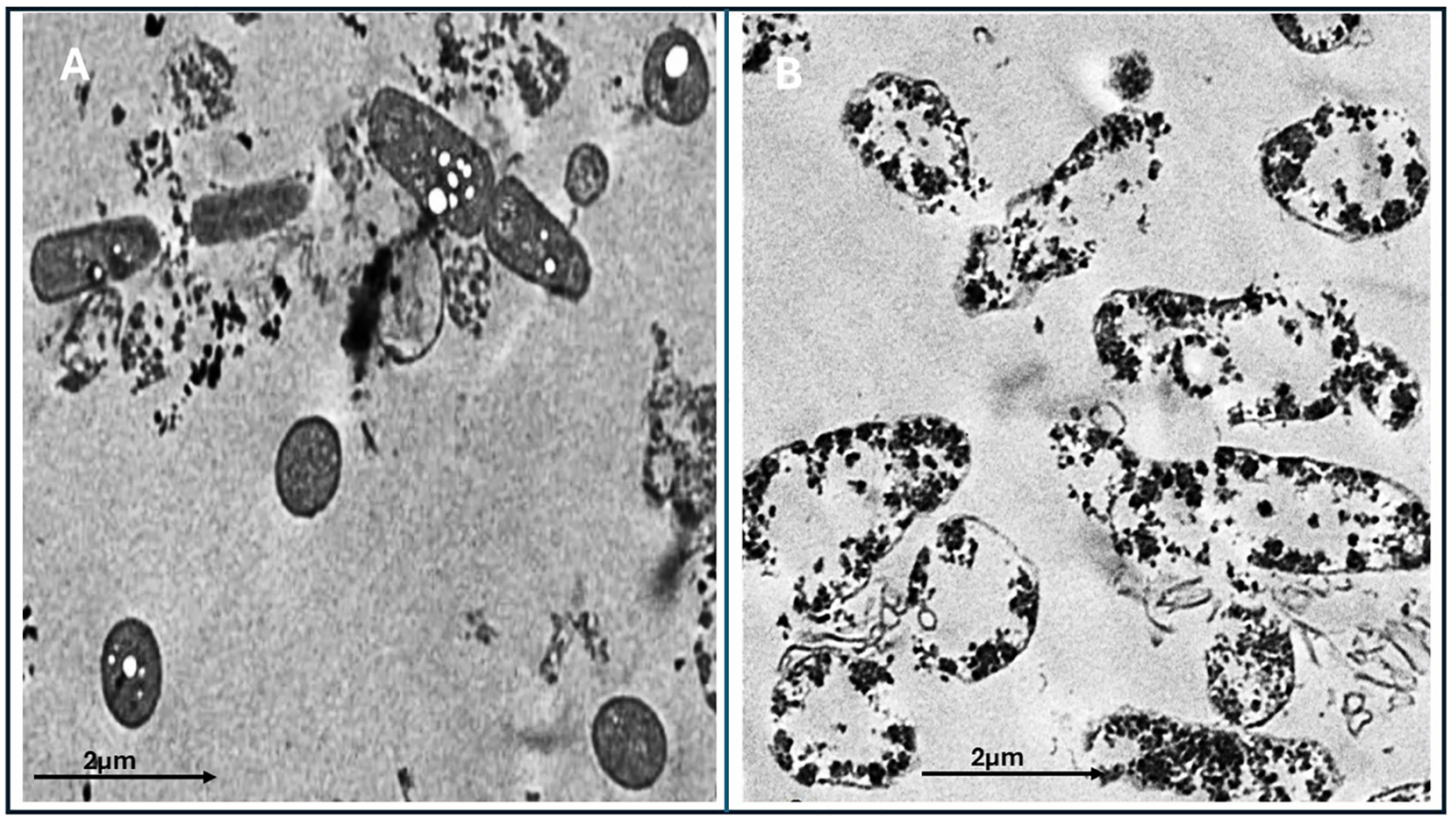
Transmission electron microscopy (TEM) images of the treated and untreated *Escherichia fergusonii* isolates. **(A)** Bacterial cells treated with garlic extract alone (control), showing relatively intact cellular structures with limited disruption. **(B)** Bacterial cells treated with biosynthesized AgNPs using garlic extract, showing extensive structural damage, aggregation of nanoparticles, and cytoplasmic disintegration. Scale bar: 2 µm.

*E. fergusonii* cells exposed solely to garlic extract exhibited minimal structural changes when examined by TEM, suggesting limited antibacterial efficacy under the experimental conditions (**Figure 5A**). In contrast, cells treated with AgNPs synthesized using garlic extract displayed substantial cellular disruption (**Figure 5B**). These included pronounced morphological deformities, such as compromised membranes, irregular shapes, and the presence of electron-dense particles indicative of nanoparticle accumulation. The internal cell architecture appeared highly disorganized and granulated, reflecting severe cellular damage and loss of integrity. The extent of ultrastructural deterioration highlights the strong antibacterial activity of garlic-mediated AgNPs, likely driven by mechanisms that impair membrane function and disrupt intracellular processes.

### Time-kill curve assays

**Figure 6:**
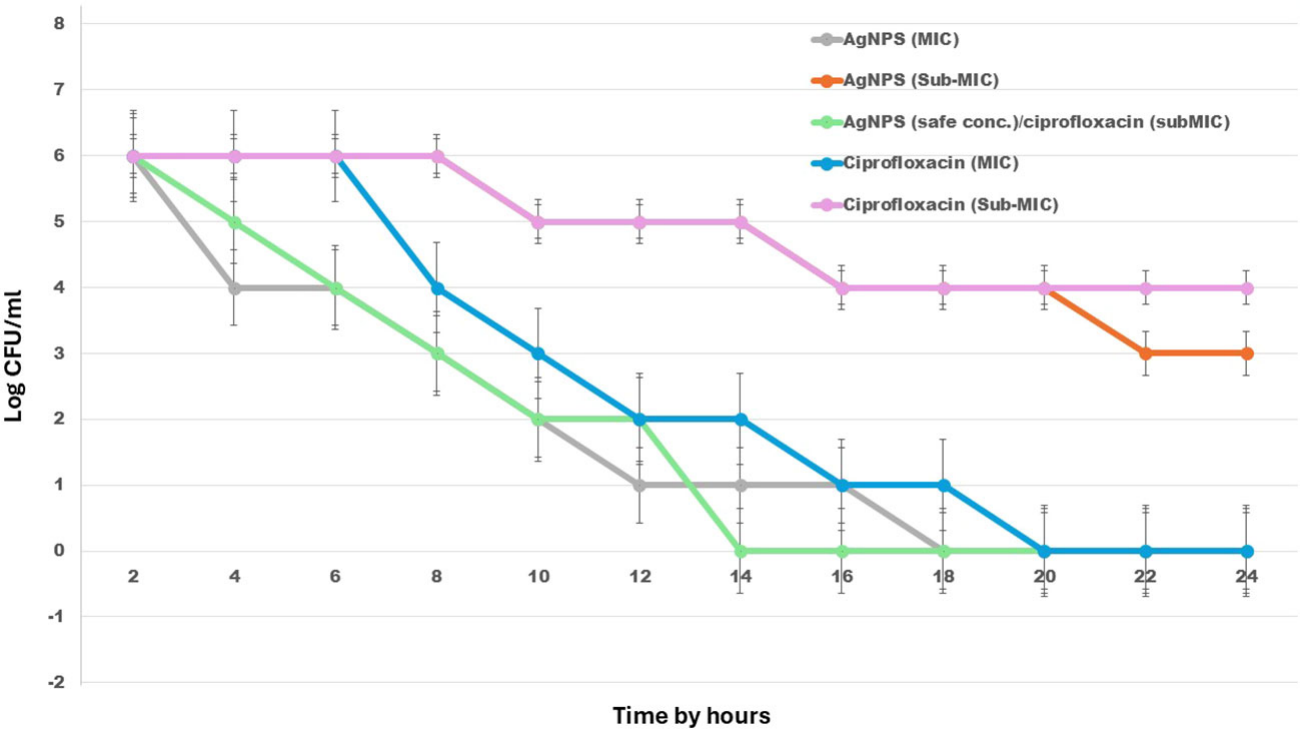
Time-kill kinetics of AgNPs, ciprofloxacin, and their combination against *E. fergusonii* over 24 hours. The graph illustrates changes in bacterial viability (log_10_ CFU/mL) in response to various treatments, including AgNPs, ciprofloxacin, and their combined application at different concentrations. .

The time-kill assay demonstrated the bactericidal efficacy of AgNPs and ciprofloxacin, both individually and in combination, against the investigated *E. fergusonii* strain over a 24-hour period. Treatment with the MIC of AgNPs (100 µg/mL) or ciprofloxacin (16 µg/mL) resulted in complete bacterial eradication by 20 hours, indicating strong individual antibacterial activity. Notably, the combination of AgNPs at a safe concentration (50 µg/mL) with ciprofloxacin at sub-MIC levels (8 µg/mL) exhibited the most rapid and potent effect, achieving total bacterial killing as early as 14 hours, highlighting a synergistic interaction. In contrast, sub-MIC treatments of either agent alone showed limited bactericidal effects and delayed reductions in bacterial viability. These results suggest that combining low concentrations of AgNPs with sub-inhibitory levels of antibiotics can significantly enhance antimicrobial efficacy (**Figure 6**).

## Discussion

Complicated wound infections remain a significant challenge in clinical settings, contributing to increased illness, longer hospital stays, and higher healthcare costs. Effective management of such infections requires a combination of prevention, early diagnosis, and proper treatment. Key preventive strategies include strict adherence to aseptic techniques, preoperative antibiotic use, good patient preparation, and careful postoperative wound care. Advances in infection control, such as antimicrobial-coated sutures and modern wound dressings, have further improved healing. However, complications may arise when standard treatment protocols fail to effectively target unusual or emerging bacteria, such as *E. fergusonii*, which may not be well-covered by common antibiotics. If an infection develops, it’s important to correctly identify the bacteria and test which antibiotics work best, so the most effective treatment can be given (Elsayed et al., 2022). In more severe cases, surgery such as cleaning the wound or draining pus may also be necessary. These challenges highlight the importance of developing new strategies alongside antibiotics to reduce wound infections and improve surgical outcomes (Pittet et al., 2009; Berríos-Torres et al., 2017). In this study, we suggest using a sublethal, safe dose of AgNPs synthesized by garlic extract in combination with antimicrobial drugs as a strategy to manage this atypical wound infection.

In our study, *E. fergusonii* emerged as a significant pathogen associated with complicated wound infections. This pathogen was consistently detected in clinical samples from patients with severe, chronic wounds, highlighting its significant role in the pathogenesis of these infections. Our findings suggest that *E. fergusonii* may play a central role in the persistence and progression of infected wounds, complicating treatment regimens and posing a substantial challenge to effective clinical management. This pathogen was among the most commonly implicated in wound infections and is known for its ability to cause severe complications in postoperative patients (Torkan and Askari Badouei, 2024; Bansal et al., 2016). Notably, all the detected *E. fergusonii* isolates exhibited multidrug-resistant (MDR) patterns, a finding consistent with the growing global concern regarding antimicrobial resistance in clinical settings (Pittet et al., 2009). The high prevalence of MDR pathogens in our study highlights the increasing difficulty in treating complicated wounds with conventional antimicrobial therapies. This emphasizes the need for innovative approaches to manage these infections, including better surveillance, rapid diagnostic methods, and the development of alternative therapies (Monk et al., 2024; Negut et al., 2018; Abd El-Hamid et al., 2023). Additionally, the polymicrobial nature of complicated wounds further complicates treatment, requiring tailored therapeutic regimens to address the diverse array of resistant pathogens present (Tatarusanu et al., 2023). Our findings underscore the critical need for multidisciplinary strategies to tackle the rising incidence of atypical MDR pathogens associated with wound infections and improve patient outcomes.

The MAR indices observed in *E. fergusonii* isolates in this study ranged from 0.56 to 0.78, indicating a high level of resistance. According to Krumperman’s threshold, MAR indices above 0.2 suggested that the bacteria originated from high-risk environments where antibiotics were frequently used (Krumperman, 1983). To better understand the clinical relevance of these values, we compared them with MAR indices reported for other common wound pathogens. For example, *Staphylococcus aureus* isolated from wound infections had shown MAR values ranging from 0.30 to 0.60 (Pai et al., 2010), while *Pseudomonas aeruginosa* had been reported with indices between 0.35 and 0.65 (Alkhulaifi and Mohammed, 2023). For instance, *Klebsiella pneumoniae* isolates from clinical specimens have exhibited MAR indices with a mean value of 0.74 (Ogefere and Idoko, 2024). The MAR indices of *E. fergusonii* in our study fell at the higher end of this spectrum, suggesting that this organism might have possessed comparable or even greater multidrug resistance potential. Given the emerging role of *E. fergusonii* as a clinically significant pathogen, particularly in wound infections, its high MAR values underscored the need for vigilant antimicrobial stewardship and further epidemiological monitoring.

The results of our study indicated that AgNPs were successfully with the most stable and uniformly sized particles exhibiting the highest antimicrobial activities at a garlic extract concentration of 10 mL (equal volume), a reaction temperature of 40°C, and a pH of 8.0. This finding is consistent with previous research demonstrating that the synthesis of AgNPs is highly influenced by the concentration of the reducing agent, temperature, and pH (Krishnan et al., 2020). This observation aligns with previous studies indicating that mildly alkaline conditions (pH 7.5–8.5) facilitate the reduction of silver ions while maintaining particle stability and functional integrity in green synthesis approaches (Ahmed et al., 2016b; Sharma et al., 2009). Garlic extract, rich in bioactive organosulfur compounds like allicin, acts effectively as both a reducing and capping agent under these conditions, helping to control nanoparticle size and prevent agglomeration (Iravani, 2014; Singh et al., 2018) and enhancing their stability and bioactivity (Vijayakumar et al., 2019). The chosen conditions therefore reflect an optimized compromise between synthesis efficiency, nanoparticle quality, and biological performance (Liaqat et al., 2022).

In the same context, the long-term and physiological stability of AgNPs is critical for their successful application in biological systems, especially for wound treatment. In this study, the garlic-synthesized AgNPs maintained colloidal stability in simulated wound fluid, a complex protein- and salt-rich environment designed to mimic wound exudate. Over 72 hours, minimal changes were observed in SPR peak position and zeta potential values, with no visible aggregation, indicating robust nanoparticle stability. These results support earlier reports that phytochemical-capped AgNPs exhibit superior stability due to their natural capping agents (Ahmed et al., 2016; Iravani, 2014). The sustained negative zeta potential (~−27 mV) observed in our study is a key indicator of electrostatic repulsion sufficient to prevent aggregation under physiological ionic strength. Stability in such a biologically relevant medium further validates the potential of these AgNPs for *in vivo* or topical wound applications, as instability could otherwise reduce efficacy or lead to rapid clearance or toxicity (Franci et al., 2015).

Our findings align with previous reports on the antimicrobial efficacy of plant-mediated AgNPs, and highlight the potential of garlic extract as a reliable bio-reductant and stabilizer. For comparison, AgNPs synthesized using *Azadirachta indica* (neem) leaf extract have demonstrated broad-spectrum antibacterial activity, particularly against *Staphylococcus aureus* and *Escherichia coli*, with inhibition zones ranging from 10 to 20 mm depending on concentration and particle size (Shankar et al., 2004). Similarly, *Curcuma longa* (turmeric)-based AgNPs have shown significant antimicrobial effects, attributed to curcumin’s polyphenolic content acting as both a reducing and capping agent (Veerasamy et al., 2011). In our study, garlic-mediated AgNPs exhibited comparable antimicrobial performance, suggesting that allicin and sulfur-containing compounds may play a synergistic role in nanoparticle synthesis and biological activity. Compared to neem and turmeric, the synthesis using garlic yielded relatively uniform nanoparticles with strong inhibition zones, indicating its potential as an efficient and cost-effective alternative for green nanoparticle production.

Of note, the antimicrobial efficacy of AgNPs is known to increase with particle size uniformity, as smaller, more stable nanoparticles often exhibit improved interaction with microbial cell membranes, leading to greater antimicrobial effects (Rai et al., 2009). These findings highlight the importance of optimizing biosynthesis conditions to achieve nanoparticles with desirable characteristics, both in terms of stability and antimicrobial potency. Our study demonstrated that AgNPs exert their antimicrobial effect primarily by interacting with microbial cell membranes and releasing ROS. The nanoparticles adhere to the bacterial cell surface, disrupting the integrity of the membrane and leading to increased permeability. This disruption allows for the leakage of essential cellular contents, such as proteins and ions, which ultimately results in cell death (Bruna et al., 2021). Additionally, AgNPs can generate ROS that further damage the microbial DNA, proteins, and lipids, compounding the antimicrobial effect (Rai et al., 2009).

This study confirmed the potent antibacterial activity of both AgNPs and ciprofloxacin against *E. fergusonii*, with each agent independently achieving complete bacterial growth inhibition at their respective MICs within 20 hours. However, the MIC of AgNPs (100 µg/mL) exceeds the cytotoxic threshold for mammalian cells, raising concerns about its clinical safety. Previous studies have shown that concentrations above 50 µg/mL can significantly affect cell viability (Ahamed et al., 2010). Notably, the combination of a lower, non-cytotoxic concentration of AgNPs (50 µg/mL) with sub-MIC ciprofloxacin (8 µg/mL) demonstrated a rapid and enhanced bactericidal effect, achieving complete bacterial inhibition within 14 hours. This synergistic interaction may be attributed to the ability of AgNPs to disrupt bacterial membranes, thereby increasing antibiotic uptake (Ibraheem et al., 2022). The limited effectiveness of sub-MIC treatments when used alone further underscores the value of combining agents to enhance efficacy while reducing potential toxicity. Overall, these findings support the potential of AgNP-antibiotic combinations as a promising strategy for managing drug-resistant or emerging pathogens more effectively and safely.

## Conclusion

Based on our findings, we recommend the use of silver nanoparticles as part of a combined treatment strategy with conventional antibiotics for managing complicated wound infections caused by both typical and atypical pathogens. AgNPs, when applied through coated dressings or wound irrigations, offer localized antimicrobial activity that can enhance the overall antibacterial effect. This combination has been shown to improve bacterial clearance, reduce the need for high systemic antibiotic doses, and potentially limit the emergence of antibiotic resistance. Integrating AgNPs with standard antibiotic therapy may therefore represent an effective and targeted approach to support wound healing and reduce post-operative complications.

## Data Availability

The datasets presented in this study can be found in online repositories. The names of the repository/repositories and accession number(s) can be found in the article/**Supplementary Material**.

## Supplementary material

Supplementary Figure 1 GC-MS chromatographic profiles of partially purified garlic extract. The figure shows the chemical fingerprint of volatile constituents present in the extract. Peaks represent individual compounds separated based on their retention times and detected by mass spectrometry.
