# Supplementary figures and images for: Eco-friendly silver nanoparticles from garlic: a novel therapeutic approach for treating Escherichia fergusonii wound infections
Source: Front Cell Infect Microbiol. 2025 Jun 30;15:1604507. doi: 10.3389/fcimb.2025.1604507 (PMC12256498; doi:10.3389/fcimb.2025.1604507)

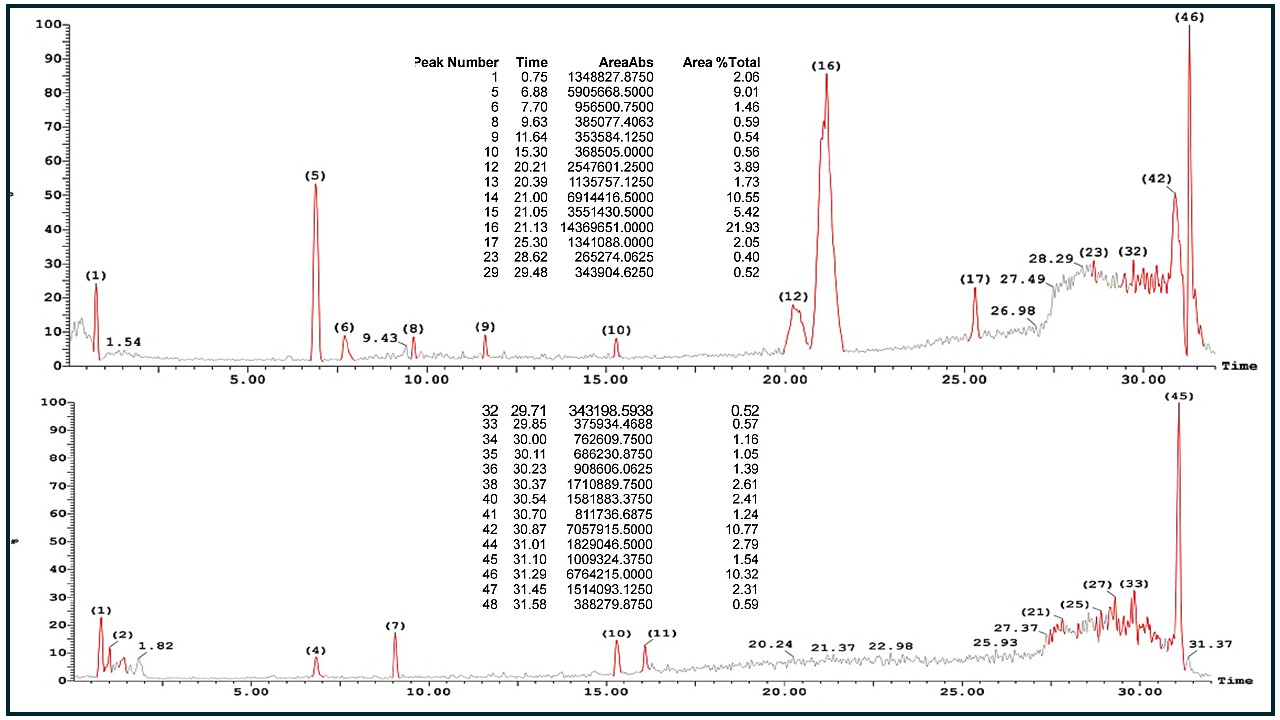

Supplement: Supplementary Figure 1 — GC-MS chromatographic profiles of partially purified garlic extract. The figure shows the chemical fingerprint of volatile constituents present in the extract. Peaks represent individual compounds separated based on their retention times and detected by mass spectrometry. [file SupplementaryFile1.jpeg]
